# Physical, Chemical, and Biological Methods for the Removal of Arsenic Compounds

**DOI:** 10.1155/2014/503784

**Published:** 2014-02-17

**Authors:** K. T. Lim, M. Y. Shukor, H. Wasoh

**Affiliations:** ^1^Department of Biochemistry, Faculty of Biotechnology and Biomolecular Sciences, Universiti Putra Malaysia (UPM), Serdang, 43400 Selangor, Malaysia; ^2^Department of Bioprocess Technology, Faculty of Biotechnology and Biomolecular Sciences, Universiti Putra Malaysia (UPM), Serdang, 43400 Selangor, Malaysia

## Abstract

Arsenic is a toxic metalloid which is widely distributed in nature. It is normally present as arsenate under oxic conditions while arsenite is predominant under reducing condition. The major discharges of arsenic in the environment are mainly due to natural sources such as aquifers and anthropogenic sources. It is known that arsenite salts are more toxic than arsenate as it binds with vicinal thiols in pyruvate dehydrogenase while arsenate inhibits the oxidative phosphorylation process. The common mechanisms for arsenic detoxification are uptaken by phosphate transporters, aquaglyceroporins, and active extrusion system and reduced by arsenate reductases via dissimilatory reduction mechanism. Some species of autotrophic and heterotrophic microorganisms use arsenic oxyanions for their regeneration of energy. Certain species of microorganisms are able to use arsenate as their nutrient in respiratory process. Detoxification operons are a common form of arsenic resistance in microorganisms. Hence, the use of bioremediation could be an effective and economic way to reduce this pollutant from the environment.

## 1. Introduction

Arsenic is one of the toxic metalloids that exists in more than 200 different mineral forms, where 60% of them are normally arsenates; 20% are sulphosalts and sulphides; and the remaining 20% are arsenite, oxides, arsenide, silicates, and elemental arsenic [1, 2]. The intrusion of orogenesis and granitic magma have resulted in the formation of arsenopyrite [1]. Arsenic was first discovered by Albertus Magnus in the year 1250 [3]. Under natural condition, arsenic normally cycled at the earth surface where the breakdown of rocks has converted arsenic sulfides into arsenic trioxide [2, 4]. Furthermore, arsenic is known to have multiple oxidation states where they are present in either organic or inorganic compounds in an aquatic environment [5, 6]. Both Zobrist et al. [7] and Root et al. [8] indicated that the mobility of arsenic inorganic compound in contaminated aquatic and sediment environment is controlled by redox processes, precipitation, sorption, and dissolution processes. It is known that ferric iron phase plays an important role for the sorption of dissolved arsenate in oxic groundwater [8]. Meanwhile, the reduction of arsenate into arsenite in the transition from aerobic to anoxic pore waters is often mediated by microbial activity, which includes detoxification and metabolic mechanisms [8]. In another study, Saalfield and Bostick [9] proposed that the presence of calcium and bicarbonate from the byproducts of biological processes in the aquifers will enhance the release of arsenic and the correlations between calcium and bicarbonate with arsenic were then observed.

Arsenic usually exists in four oxidation states: As^−3^ (arsine), As° (arsenic), As^+3^ (arsenite), and As^+5^ (arsenate) [4, 10]. In soil environment, arsenic is generally present in two oxidation states which are As^+3^ (arsenite) and As^+5^ (arsenate) and normally present as a mixture of As^+3^ (arsenite) and As^+5^ (arsenate) in air [2]. Of the two oxidation states, arsenate is the main species associated with soil arsenic contaminations, and it is often written as AsO_4_^3−^ which is very similar to phosphate [11, 12]. Arsenate could act as a potential oxidative phosphorylation inhibitor. This is a cause for concern since oxidation phosphorylation is the main key reaction of energy metabolism in humans and metazoans [4]. Arsenite has been reported as the most toxic and soluble form of arsenic when compared to arsenate, and it can bind with reactive sulfur atoms present in many enzymes, including enzymes which are involved in respiration [4, 13]. Furthermore, it is known that soluble inorganic arsenic is often more toxic than the organic form [2]. Unlike arsenate and arsenite, arsine is often available as highly toxic gases such as (CH_3_)_3_ and H_3_As and often present at low concentration in the environment [4].

Meanwhile, the average concentration of arsenic in fresh water is around 0.4 *μ*g/L and could reach 2.6 *μ*g/L in seawater [13]. However, the thermal activity in some places has caused high level of arsenic in waters with the concentration of arsenic in geothermal water in Japan ranging from 1.8 to 6.4 mg/L whereas the concentration of arsenic in New Zealand water could reach up to 8.5 mg/L [2, 29, 30]. In extreme cases, analysis from well drinking water in Jessore, Bangladesh, revealed that the levels of arsenic could reach up until 225 mg/L [31]. On the other hand, the concentration of arsenic in plants is solely depending on the amount of arsenic that the plant is being exposed to where the concentration of arsenic could range from less than 0.01 *μ*g/g (dried weight) in the uncontaminated area to around 5 *μ*g/g (dried weight) in the contaminated area [2]. Unlike plant, the concentration of arsenic in marine organisms and mammals has a wide range of variations ranging from 0.005 to 0.3 mg/kg in some crustaceans and molluscs, 0.54 *μ*g/g in fish, over 100 *μ*g/g in some shellfish, and less than 0.3 *μ*g/g in humans and domestic animals [2]. Presence of humic acid in the shallow subsurface could affect the mobility of arsenic since humic acid could interact with aqueous arsenic for the formation of stable colloidal complexes that might play a prominent role in the enhancement of arsenic mobility. Furthermore, the combination of humic acid together with ferric hydroxide surface will lead to the formation of stable complexes that would compete with arsenic for its adsorption sites [32].

## 2. Usage of Arsenic

The first usage of arsenic in medicine could be dated around 2500 years ago where it was mainly consumed for the improvement of breathing problems as well as to give freshness, beauty, and plumpness figures in women [2]. Arsenic in the form of arsenical salvarsan (arsenic containing drug) was the initial antimicrobial agent used in the treatment of infectious diseases such as syphilis and sleeping sickness in 1908 [3]. This drug was specifically developed by Sahachiro Hata under the guidance of Paul Ehrlich in 1908 where they named the drug as arsphenamine no. 606 [33]. Meanwhile, arsenic in the form of arsenic trioxide (As_2_O_3_) is one of the most common forms of arsenic, which is often used in manufacturing and agriculture industry and for medical purposes such as in the treatment of acute promyelocytic leukemia [34]. Arsenic trioxide is also proven to be useful in criminal homicides due to its characteristic, which is tasteless, colorless, highly toxic, and soluble in water [2, 4]. The high usage of arsenic trioxide in suicide cases had made it to be often referred as the “inheritance powder” in the 18th century [4].

During the 1970s, arsenic was mainly used in agriculture industry in the form of insecticide's component in order to get rid of the insects [2, 13, 35]. Arsenic was also used as cotton desiccants and wood preservatives in United States [2]. The usage of arsenic as the cotton desiccant was introduced around year 1956 and was widely used due to its effectiveness and affordable price [36]. Besides that, arsenic was also being used in ceramic and glass industry, pharmaceutical industry, and food additives as well as pigments in paint [13, 34]. Meanwhile, arsenic in the form of 4-aminoben-zenearsenic acid (p-arsenilic acid, p-ASA) has been used as animals food additive for feeding of boiler chickens [37].

## 3. Toxicity of Arsenic

It has been noticed that the extensive usage of arsenic in the industrial and agrochemical applications is of few causes of groundwater and sediment arsenic contamination in the environment [6, 38] in which effects are much smaller compared to the natural causes [39]. The presence of arsenic in soil and water has become an increasing problem in many countries around the world, including Bangladesh, India, Chile, and Taiwan [2, 40, 41], and natural geological source is one of the main causes of contamination [34]. Consumption of drinking water that has been contaminated by hazardous level of arsenic will lead to a wide range of diseases such as arsenic dermatosis, lung cancer, liver cancer, uterus cancer, skin cancer and occurrence of skin, and bladder and hepatocellular carcinoma that will result in slow and painful death [1, 2, 41–43]. In Southwestern Taiwan, the human consumption of artesian well waters which contains high concentration of arsenic has also led to Blackfoot disease, which is an endemic peripheral vascular disease in that area [40]. In China, up to the year 2012, 19 provinces had been found to have As concentration in drinking water exceeding the standard level (0.05 mg/L). Inner Mongolia, Xinjiang, and Shanxi Provinces are historical well-known “hotspots” of geogenic As-contaminated drinking water [44].

Deltaic plain contaminated groundwater of Ganges-Meghna-Brahmaputra rivers in Bangladesh and West Bengal had resulted in an alarming environmental problem as this water is often consumed by people who live in that area [6, 45]. The presence of aqueous arsenic is mainly due to rock weathering as well as sediment deposition and downstream transport of rich mineral arsenic that was originally present in Himalayas [4]. Massive constructions of wells which are meant to supply an improved quality of water with waterborne pathogens free to the people living in this area had created another problem as the ground water in that area was arsenic contaminated [4]. In Nepal, arsenic (As) contamination was a major issue in water supply drinking systems especially in high density population such as Terai districts. The local inhabitants still use hand tube and dug wells (with hand held pumps that are bored at shallow to medium depth) for their daily water requirements [46]. The results of the analysis on 25,058 samples tested in 20 districts, published in the report of arsenic in Nepal, demonstrated that 23% of the samples were containing 10–50 *μ*g/L of As, and 8% of the samples were containing more than 50 *μ*g/L of As. Recent status from over 737,009 samples tested has shown that 7.9% and 2.3% were contaminated by 10–50 *μ*g/L and >50 *μ*g/L of As, respectively [46]. Other places reporting the ground water arsenic contamination include south West Coast of Taiwan; Antofagasta in Chile; six areas of Region Lagunera located in the central part of North Mexico; Monte Quemado; Cordoba province in Argentina; Millard County in Utah, United States; Nova Scotia in Canada; and Inner Mongolia, Qinghai, Jilin, Shanxi, Xinjiang Uygur A.R., Ningxia, Liaoning, and Henan provinces in China [2].

Accidental ingestion of pesticides or insecticides containing arsenic will also result in an acute arsenic poisoning which sometimes could lead to mortality when 100 mg to 300 mg of doses were being consumed [1]. The symptoms of acute arsenic poisoning are vomiting, abdominal pain, diarrhea, and cramping, which will then cause renal failure, haematological abnormalities such as leukemia and anemia, pulmonary oedema, and respiratory failure, and it could further lead to shock, coma, and death [1, 2, 34]. In another study, Lai et al. [47] reported that the consumption of water contaminated with arsenic will increase the risk of diabetes mellitus by twofold. In US, prevalence of diabetes increased among people having urine arsenic concentrations in more than 20% of the general population [48]. Arsenic contamination from industrial sources has also led to skin manifestation of chronic arsenic poisoning, which affected 19.9% of the human populations living in Ron Phibun, Thailand [2]. On the other hand, arsenic poisoning caused by ingestion of food (especially seafood product) and beverages contaminated by arsenic has been reported in Japan, England, Germany, and China [2]. In Campinas, Brazil, 116 samples of seafood (used for sashimi making) from Japanese restaurants have been evaluated for the presence of As [49]. Several samples were found with percentage above the maximum limit permitted by European regulations including 90% tuna, 48% salmon, 31% mullet, and 100% octopus. It was concluded that the octopus was the sashimi which most contributed to arsenic. In other case, the arsenic concentration in rice was found to be high in Bangladesh [50].

Phosphate fertilization is suggested to lower the arsenate uptake in plants because both compounds enter the rice via the same transporter. However, there are arguments in certain cases because under flooding conditions, As is present as arsenite, which cannot compete with phosphate; furthermore, phosphate increases As mobility because it competes with arsenate for the adsorption site on Fe-oxides/hydroxides [51]. Presence of over 1.0 *μ*g/g arsenic concentration in hair, 20 to 130 *μ*g/g in nails, and over 100 *μ*g per day in urine is an indication of arsenic poisoning [2]. Significant correlation was also observed with levels in human urine, toenail, and hair samples [31]. A meta-analysis assessing the effects of exposure to arsenic suggests that 50% increment of arsenic levels in urine would be associated with 0.4 decrement in the intelligence quotient (IQ) of children aged 5–15 years [52]. Arsenic uptake is adventitious because arsenate and arsenite are chemically similar to the required nutrients [53]. At neutral pH, the trivalent forms of these metalloids are structurally similar to glycerol, and hence they can enter cells through aquaporins [54].

## 4. Technologies/Methods for the Removal of Arsenic from Environment

According to World Health Organisation (WHO) standard set in the year 1993, the maximum limit of arsenic contamination in drinking water is 10 *μ*g/L or 10 ppb. [1]. This limit was later adopted by European Union in the year 1998 (council directive 98/83/EC), transposed by Portuguese legislation by Law Decree (DL) number 236/2001 [1, 55]. In the year 2006, United States has also adopted the WHO standard for lowering the federal drinking water standard for maximum limit of arsenic from 50 *μ*g/L to 10 *μ*g/L [8]. Technologies for removing arsenic from the environment should meet several basic technical criteria that include robustness, no other side effect on the environment, and the ability to sustain water supply systems for long terms and meet the quality requirement of physical chemical, and microbiological approaches [1]. Currently, there are many methods for removing arsenic from the soil contaminated with arsenic, which could be divided into three categories, including physical, chemical, and biological approaches [14].

In the physical approaches, the concentration of arsenic in soil could be reduced by mixture of both contaminated and uncontaminated soils together that will lead to an acceptable level of arsenic dilution [14]. Soil washing is another method which is grouped under physical approaches whereby arsenic contaminated soil will be washed with different concentration of chemicals such as sulfuric acid, nitric acid, phosphoric acid, and hydrogen bromide [14]. The choice of chemicals used for extractant and high cost have often restricted the usage of soil washing into a smaller-scale operations as it is the disadvantages of using soil washing method [14]. Meanwhile, cement can immobilise soluble arsenites and has been successfully used to stabilise As-rich sludges which may be suitable for treating sludges generated from precipitative removal units [15]. Furthermore, the disposal of water treatment wastes containing As, with a particular emphasis on stabilisation/solidification (S/S) technologies, has been assessed for their appropriateness in treating As containing wastes. In this process, brine resulting from the regeneration of activated alumina filters is likely to accelerate cement hydration. Furthermore, additives (surfactants, cosolvents, etc.) have also been used to enhance the efficiencies of soil flushing using aqueous solutions as water solubility is the controlling mechanism of contaminant dissolution. The usage of surfactant alone gives about 80–85% of efficiencies in laboratory experiments. Studies indicated that when soil flushing is applied in the field, efficiency can vary from 0% to almost 100%. It often gives moderate efficiencies by using only one product (surfactant, cosolvent, and cyclodextrin). On the other hand, the use of more complex methods with polymer injection leads to higher efficiencies [16].

The current available chemical remediation approaches mainly involving methods such as adsorption by using specific media, immobilization, modified coagulation along with filtration, precipitations, immobilizations, and complexation reactions [1, 14]. The coagulation along with filtration method for removing arsenic from contaminated sources is quite economic but often displayed lower efficiencies (<90%) [1]. The formation of stable phases, for example, insoluble FeAsO_4_ (and hydrous species of this compound such as scorodite, FeAsO_4_·2H_2_O), is beneficial for the stabilization procedure [17]. Furthermore, the use of selective stabilizing amendments is a challenging task as the majority of polluted sites are contaminated with multiple metal(loid)s. Nanosized oxides and Fe(0) (particle size of 1 to 100 nm) are another possible enhancement for the stabilization method [17]. Natural nanoparticulate oxides are important scavengers of contaminants in soils [56] and due to their reactive and relatively large specific surface area (tens to hundreds m^2^/g), engineered oxide nanoparticles are promising materials for the remediation of soils contaminated with inorganic pollutants [18, 19]. It is reported that chemical remediation gained popularity because of its high success rate, but it could be expensive when someone would like to remediate a large area [14]. In contrast, biological remediation or bioremediation of soils contaminated with either inorganic or organic arsenic present in pesticides and hydrocarbons have been widely accepted in some places [14]. Even though bioremediation suffers several limitations, these approaches have been gaining interest for the remediation of metal(loid) contaminated soils due to their cost effectiveness [14]. Basically, bioremediation technology could be divided into subcategories: intrinsic bioremediation and engineered bioremediation [14]. Intrinsic bioremediation is generally referred to as the degradation of arsenic by naturally occurring microorganisms without intervention by human, and this method is more suitable for remediation of soil with a low level of contaminants [14]. Engineered bioremediation often relies on intervention of human for optimizing the environment conditions to promote the proliferation and activity of microorganisms that lived in that area. As a result, the usage of engineered bioremediation method is more favorable in the highly contaminated area [14].

Mechanism for arsenic detoxification can be divided into four which known as uptake of As(V) in the form of arsenate by phosphate transporters, uptake of As(III) in the form of arsenite by aquaglyceroporins, reduction of As(V) to As(III) by arsenate reductases, and extrusion or sequestration of As(III) [57]. AQPs have been shown to facilitate diffusion of arsenic [53, 54]. The microbial oxidation of As in Altiplano basins (rivers in northern Chile) was demonstrated by Leiva et al. [20]. The oxidation of As (As(III) to As(V)) is a critical transformation [58] because it favors the immobilization of As in the solid phase. As(III) was actively oxidized by a microbial consortium, leading to a significant decrease in the dissolved As concentrations and a corresponding increase in the sediment's As concentration downstream of the hydrothermal source. In situ oxidation experiments demonstrated that the As oxidation required a biological activity, and microbiological molecular analysis had confirmed the presence of As(III)-oxidizing groups (aro A-like genes) in the system. In addition, the pH measurements and solid phase analysis strongly suggest that As removal mechanism must involve adsorption or coprecipitation with Fe-oxyhydroxides. Taken together, these results indicated that the microorganism-mediated As oxidation contributed to the attenuation of As concentrations and the stabilization of As in the solid phase, therefore controlling the amount of As transported downstream [20]. Since most of the cases of arsenic poisoning are due to the consumption of water contaminated by arsenic, the process of cleaning up or reducing arsenic concentration in water becomes very important. Methods used in reducing arsenic levels in water are primarily divided into (i) physiochemical methods, which include filtration or coagulation sedimentation, osmosis or electrodialysis, adsorptions, and chemical precipitations and (ii) biological methods such as phytoremediation by using aquatic plants or microbial detoxification of arsenic [14].

Generally, two approaches are mainly employed in the phytoremediation method. The first approach uses “free-floating plants such as water hyacinth” that could adsorb metal(loid)s and the plants would be removed from the pond once the equilibrium state is achieved [14]. The second approach uses aquatic rooted plants (i.e., *Agrostis *sp., *Pteris vittata, Pteris cretica,* and others) to remove arsenic from bed filters and from water [14, 21–23]. Yang et al. [23] stated that the addition of arsenate reducing bacteria will promote the growth of *P. vittata* in soil. Two important processes in the removal of arsenic from water by microorganisms are biosorption and biomethylation [14]. It is reported that biomethylation (by As(III) *S*-adenosylmethionine methyltransferase) is the reliable biological process for removing arsenic from aquatic media [14].

Recently, the arsenite (As(III)) *S*-adenosylmethionine methyltransferase (ArsM) gene has been inserted into the chromosome of *Pseudomonas putida* KT2440 for potential bioremediation of environmental arsenic [59]. The first structure of As(III) *S*-adenosylmethionine methyltransferase by X-ray crystallography was described by [60]. In this enzyme, there are three conserved cysteine residues at positions 72, 174, and 224 in the CmArsM orthologue from the thermophilic eukaryotic alga *Cyanidioschyzon* sp. 5508 [61]. Substitution of any of the three led to the loss of As(III) methylation [61]. The relationship between the arsenic and *S*-adenosylmethionine binding sites to a final resolution of ~1.6 Å. As(III) binding causes little change in conformation, but binding of SAM reorients helix *α*4 and a loop (residues 49–80) towards the As(III) binding domain, positioning the methyl group to be transfer to the metalloid [60].

## 5. Arsenic Resistant Microorganisms 

Studies of bacterial growth at high arsenic-phosphorus ratios demonstrated that high arsenic concentrations can be tolerated relatively and that it can be involved in vital functions in the cell [62]. *Corynebacterium glutamicum* survives arsenic stress with two different classes of arsenate reductases. Cg-ArsC1 and Cg-ArsC2 are the single-cysteine monomeric enzymes coupled to the mycothiol/mycoredoxin redox pathway using a mycothiol transferase mechanism, while Cg-ArsC1' is a three-cysteine containing homodimer that uses a reduction mechanism linked to the thioredoxin pathway [63]. The presence of naturally occurring arsenate and arsenite in water and soil environment which could enter the cells by the phosphate-transport system has given pressure for microorganisms to maintain their arsenic detoxification systems for surviving purposes. One of the commonest forms of arsenic resistance in microorganisms is by detoxification operons, which are encoded on genomes or plasmids [64].

Most of the detoxification operons consist of three genes, which are known as *arsC* (reduction of arsenate to arsenite), *arsR* (transcriptional repressor), and *arsB* (can also be a subunit of the ArsAB As(III)-translocating ATPase, an ATP-driven efflux pump) [3, 53, 64]. Moreover, some detoxification operons also contain two additional genes (*arsD-*metallochaperone and *arsA*-ATPase) [3, 64]. The ArsD metallochaperone binds cytosolic As(III) and transfers it to the ArsA subunit of the efflux pump [53]. In normal process, arsenate that enters the cell will be reduced to arsenite by *ArsC* gene before it is transported out of the cell by *ArsB* gene [10, 64]. As a result, a more toxic form of arsenic will be introduced into the environment. In another study, Villegas-Torres et al. [65] indicated that the arsenic resistance ability in *Bacillus sphaericus* could be due to the presence of *arsC* gene, which could be horizontally transferred between microorganisms isolated from Columbian oil polluted soil that contain high arsenic levels.

The other reduction of arsenate to arsenite by microorganisms is via dissimilatory reduction mechanism that could be carried out in facultative anaerobe or strict anaerobe condition with the arsenate acting as the terminal electron acceptor [24]. These microorganisms have the ability to oxidize inorganic (sulfide and hydrogen) and organic (e.g., formate, aromatics, and lactase acetate) as an electron donor which will lead to the production of arsenite, and they were normally named dissimilatory arsenate-respiring prokaryotes (DARPs) [4].

Two other families of arsenate reductase are known as thioredoxin (Trx) clade and Arr2p arsenate reductase. It is reported that Trx clade is linked with *arsC* arsenate reductase gene while Arr2p is related to different class of larger protein tyrosine phosphatases [66]. Zargar et al. [67] reported that *ArxA* (arsenite oxidase) enzymes, which are present in *Alkalilimnicola ehrlichii* MLHE-1 strain (a chemoliautotroph bacteria) could couple with arsenite oxidation as well as nitrate reduction. Different types of bacteria with the ability of resisting arsenic are *Rhodococcus, Arthrobacter, Acinetobacter, Agrobacterium, Staphylococcus, Escherichia coli, Thiobacillus, Achromobacter, Alcaligene, Pseudomonas, Microbacterium oxydans, Ochrobactrum anthropi, Cupriavidus, Desulfomicrobium, Cyanobacteria, Sulfurospirillum, Wolinella, Citrobacter, Agrobacterium*, an arsenic reducing bacteria from* Flavobacterium-Cytophaga *group, *Scopulariopsis koningii, Fomitopsis pinicola, Penicillium gladioli, Fusarium oxysporum meloni, Fucus gardneri, Bosea *sp., *Psychrobacter *sp., *Polyphysa peniculus, Methanobacterium, Bradyrhizobium, Rhodobium, Sinorhizobium,* and *Clostridium* [13, 21, 53, 68–71].

Liao et al. [69] reported that 11 arsenic reducing bacteria strains from seven different genera (i.e., *Pseudomonas, Psychrobacter, Citrobacter, Bacillus, Bosea, Vibrio,* and *Enterobacter*) were isolated from environmental groundwater samples collected from well AG1 in Southern Yunlin County, west-central Taiwan. In Liao et al. [69] report, they indicated that diverse community of microorganisms holds a significant impact in the biotransformation of arsenic that is present in the aquifer, and these communities of bacteria are well adapted to high arsenic concentrations that are present in the water. In another study, Mumford et al. [72] reported that *Alkaliphilus oremlandii* and ferum reducing bacteria such as *Geobacter* species were present in arsenic rich groundwater beneath a site-specific site (C6) on Crosswicks Creek, New Jersey. Other bacteria with the ability of reducing arsenate to arsenite are *Sulfurospirillum barnesii *and *Sulfurospirillum arsenophilum* from the *ε*-proteobacteria as well as *Pyrobaculum arsenaticum *from Thermoproteales order and *Chrysiogenes arsenatis* [4, 10, 73, 74]. Afkar [10] reported that the reduction of arsenate to arsenite by *S. barnesii* strain SeS-3 is associated with the membrane cell where this resistance mechanism is encoded by a single operon that consists of arsenite ion-inducible repressor. Besides that, Afkar [10] also indicated that *S. barnesii* strain SeS-3 reduced arsenate to arsenite under anaerobic condition using arsenate as terminal electron acceptors while lactate as the carbon source.

In another study, Youssef et al. [75] reported that both *Neisseria mucosa* and *Rahnella aquatilis* are able to reduce arsenate and selenate. In their study, both *N. mucosa* and *R. aquatilis* were grown in a neutral pH medium (pH 7) containing five mM sodium arsenates where the sodium lactate acts as an electron donor while *N. mucosa* and *R. aquatilis* act as the electron acceptor organisms. Although both *N. mucosa* and *R. aquatilis* strains studied are able to grow at higher pH medium (pH10), their growth rate decreased drastically (reduction of 43% in *N. mucosa* and 67.2% in *R. aquatilis*) and has been observed [75]. Meanwhile, archaebacterium *Sulfolobus acidocaldarius* strain BC, *Alcaligenes faecalis, Shewanella algae*, *β*-proteobacteria strain UPLAs1, *Alcaligenes faecalis*, *Comamonas terrae* sp. nov, some heterotrophic bacteria (*Herminiimonas arsenicoxydans*), and chemoliautotrophic bacteria are reported to have the ability to oxidize arsenite to a less toxic arsenate [4, 14, 25–28]. In this case, arsenite will often serve as an electron donor for reducing the nitrate or oxygen that will produce energy in order to fix carbon dioxide [26]. Two genes, *aoxA *and *aoxB *encoding for arsenite oxidase played an essential role in the oxidation of arsenite into arsenate [27]. The insertion of mini-Tn*5::lacZ2* transposon in *aoxA *or *aoxB* gene will stop to arsenite oxidation process [27].

In another review by Silver and Phung [12], they indicated that both *asoA* and *asoB* genes which encoded for large molybdopterin-containing and small Rieske (2Fe-2S) cluster the subunit of oxidation of arsenite in *Alcaligenes faecalis*. They identified that the upstreams of *asoB* consist of 15 genes while the downstreams of *asoA *consist of six genes, which are involved in arsenic resistance and metabolisms [12]. Besides bacteria, certain species of algae such as *Fucus gardneri* and *Chlorella vulgaris* are also known to have the ability to accumulate arsenic [76, 77].

Table 1 shows the advantages and disadvantages of physical, chemical, and biological methods for the removal of arsenic compounds. Physical method exhibits the simplest choice, but it was however limited to small scale operations. Chemical method had gained popularity by its high success rate; however, the remediation area can be exposed to other types of chemical contaminants. The usage of biological and phytoremediation methods might be the most practical methods for a small area but more research needs to be carried out especially in methylations, reduction, and oxidation using microorganisms for more effective method to remove the arsenic compound as they have a high potential application in the future.

Previously, bacterial biosensors (whole-cell) were being used to detect inorganic arsenic [78]. Biosensor technology was widely studied by using potentiometry, amperometry, and conductometry [79–81]. Only a few studies were carried out based on capacitometry [82] especially by using DNA and antibodies [83, 84]. Previously, capacitive sensor using enzyme was introduced for toxin detection [82]. A biosensor selective for the trivalent organoarsenicals methyl arsenate and phenyl arsenite over inorganic arsenite was reported by Chen et al. [78] which may be useful for detecting degradation of arsenic-containing herbicides and growth promoters. A surface plasmon resonance biosensor for the study of trivalent arsenic was also reported by Liu et al. [85]. This biosensor indicates that the 3D hydrogel-nanoparticle coated sensors exhibited a higher sensitivity than that of the 2D AuNPs decorated sensors. It was shown that binding of As(III) into ArsA was greatly facilitated by the presence of magnesium ion and ATP. For future research, biosensor based on capacitometry using enzymes [82] such as As(III) *S*-adenosylmethionine methyltransferase for arsenic detection remains interesting to be explored.

## 6. Conclusion

Arsenic is a metalloid that causes harm to humans and environments. However, certain species of prokaryotes have the abilities to use arsenic either through oxidation or reduction process for energy conservation and growth purposes. It is important to remove and reduce this pollutant from the environment through different approaches such as physical, chemical, and biological. The use of bioremediation to remove and mobilize arsenic from contaminated soils and aquifers could be an effective and economic way since a wide range of microorganisms have been found to be successfully degrading this pollutant from the environment.

## Figures and Tables

**Table 1:** Advantages and disadvantages of methods for the removal of arsenic compounds.

Method	Method in Detail	Advantages/Disadvantages	Reference
Physical approaches	Mixing both contaminated and uncontaminated soils	High cost/usage to smaller-scale operations	[14]

Physical approaches	Washed with sulfuric acid, nitric acid, phosphoric acid, and hydrogen bromide	Chemicals usage/high cost/usage to smaller-scale operations	[14]

Physical approaches	Immobilise soluble arsenites using cement	Successfully used to stabilise As-rich sludges	[15]

Physical approaches	Emphasis on stabilisation/solidification (S/S)	Treating As containing wastes in water	[15]

Physical approaches	Soil flushing using aqueous solutions using surfactants and cosolvents	Applied in the field, efficiency can vary from 0% to almost 100%	[16]

Chemical remediation approaches	Adsorption by using specific media, immobilization, modified coagulation along with filtration, precipitations, immobilizations, and complexation reactions	Economic but often displayed lower efficiencies (<90%)	[1, 14]

Chemical remediation approaches	Formation of stable phases, for example, insoluble FeAsO_4_ (and hydrous species of this compound such as scorodite, FeAsO_4_.2H_2_O)	Use of selective stabilizing amendments is a challenging task	[17]

Chemical remediation approaches	Stabilization method using nanosized oxides and Fe(0) (particle size of 1 to 100 nm)	Gained popularity/high success rate, but it could be expensive when remediating a large area	[14, 17–19]

Intrinsic bioremediation	Degradation of arsenic by naturally occurring microorganisms	More suitable for remediation of soil with a low level of contaminants	[14]

Engineered bioremediation	Optimizing the environment conditions to promote the proliferation and activity of microorganisms	Favorable method used in high contaminated area	[14]

Microbial oxidation	Immobilization of As in the solid phase	Required biological activity, and microbiological molecular analysis/involved adsorption or coprecipitation with Fe-oxyhydroxides.	[20]

Physiochemical methods	Filtration or coagulation sedimentation, osmosis or electrodialysis, adsorptions, and chemical precipitations	Widely accepted in some places	[14]

Biological methods	Such as phytoremediation by using aquatic plants or microbial detoxification of arsenic	Widely accepted in some places	[14]

Phytoremediation method	Using “free-floating plants such as water hyacinth”	Widely accepted in some places	[14, 21–23]
	Using aquatic rooted plants such as *Agrostis *sp*., Pteris vittata, *and* Pteris cretica *		

Methylations	Biomethylations (by As(III) *S*-adenosylmethionine methyltransferase)	Is a reliable biological process of removing arsenic from aquatic mediums	[14]

Reduction	Reduction of arsenate into arsenite by microorganisms via dissimilatory reduction mechanism	Should be carried out in facultative anaerobe or strict anaerobe condition	[24]

Oxidation	Using heterotrophic bacteria and chemoautotrophic bacteria to oxidize arsenite into a less toxic arsenate	Should be carried out in controlled environment	[4, 14, 25–28]
